# FOXM1 promotes invasion and migration of colorectal cancer cells partially dependent on HSPA5 transactivation

**DOI:** 10.18632/oncotarget.8419

**Published:** 2016-03-28

**Authors:** Xiaoyong Luo, Jinke Yao, Peipei Nie, Zhiyuan Yang, Hongbo Feng, Pinjia Chen, Xinpeng Shi, Zhengzhi Zou

**Affiliations:** ^1^ Department of Oncology, The Affiliated Luoyang Central Hospital of Zhengzhou University, Luoyang, China; ^2^ MOE Key Laboratory of Laser Life Science and Institute of Laser Life Science, Joint Laboratory of Laser Oncology with Cancer Center of Sun Yat-sen University, College of Biophotonics, South China Normal University, Guangzhou, China; ^3^ Department of General Surgery, Boji-Affiliated Hospital (Zengcheng People's Hospital), Sun Yat-Sen University, Guangzhou, China; ^4^ KingMed Diagnostics and KingMed School of Laboratory Medicine, Guangzhou Medical University, Guangzhou, China; ^5^ Department of Medcine, The Affiliated Luoyang Central Hospital of Zhengzhou University, Luoyang, China

**Keywords:** FOXM1, HSPA5, migration, invasion, colorectal cancer

## Abstract

In this study, to investigate whether endoplastic reticulum (ER) stress correlated with FOXM1 in colorectal cancer, we analysed the mRNA levels of *FOXM1* and ER stress markers *HSPA5* and spliced *XBP1* by qRT-PCR. *FOXM1* mRNA levels were found to positively correlate with *HSPA5* in colorectal cancer. However, no significant correlation between *FOXM1* and spliced *XBP1* mRNA levels was found. Theses results suggested the positive correlation between FOXM1 and HSPA5 in colorectal cancer was not associated with ER stress. Next, we provided evidences that FOXM1 promoted HSPA5 transcription by directly binding to and stimulating HSPA5 promoter. Moreover, a FOXM1-binding site mapped between -1019 and -1012 bp of the proximal *HSPA5* promoter was identified. In addition, we found that enhancement of cell migration and invasion by FOXM1 was significantly attenuated by depletion of HSPA5 in colorectal cancer cell. Furthermore, FOXM1 triggered colorectal cancer cell migration and invasion was involved in activities of cell-surface HSPA5. Lastly, our results suggested FOXM1 facilitated the activities and expressions of MMP2 and 9 associated with cell-surface HSPA5 in colorectal cancer cells. Moreover, statistically significant positive correlations between *FOXM1* and *MMP2* mRNA expression, between *HSPA5* and *MMP2* were found in colorectal cancer tissue specimens. Together, our results suggested that FOXM1-HSPA5 signaling might be considered as a novel molecular target for designing novel therapeutic regimen to control colorectal cancer metastasis and progression.

## INTRODUCTION

Colorectal cancer is one of the most commonly occurring tumors and a prevalent cause of cancer-related death worldwide [1]. In most colorectal cancer patients, tumor is diagnosed at advanced stage accompanied by the spread of tumor cells to distant organs [2]. Successful therapeutic strategies are limited in these patients with cancer cells metastasis. Thus, investigations into the molecular mechanisms involving in colorectal cancer metastasis may help to develop some novel therapeutic regimens for targeted therapy.

*FOXM1* known as *HNF-3*, *HFH-11*, *MPP2*, *Win* and *Trident*, is a member of the forkhead box transcription factor family. The human FOXM1 protein has three isoforms FOXM1a, FOXM1b, and FOXM1c [3]. Previous reports show FOXM1 promotes tumorigenesis and is widely overexpressed in a multitude of human solid tumors, including colorectal cancer [4–6]. FOXM1 is required for regulating the transition from G1 to S and G2 to M phase in mitosis [7, 8]. Accordingly, FOXM1 serves as a dominant role in proliferation of cancer cells. On the contrary, downregulation of FOXM1 expression leads to cell cycle arrest, chromosome misaggregation and spindle defects in cancer cells [9]. In addition, FOXM1 is found to increase resistance to chemotherapy drugs in multiple types of cancer [10]. Furthermore, increasing evidence has indicated that FOXM1 plays an important role in the metastasis of several malignances including colorectal, pancreatic, ovarian and breast cancer [5, 6, 11, 12]. Studies have shown that enhanced FOXM1 levels promote cancer cell invasion and metastasis by inducing epithelial-to-mesenchymal transition (EMT) [12]. Additionally, FOXM1 has been found to increase colorectal cancer migration and invasion by regulating a various signal pathways, such as urokinase-type plasminogen activator receptor (uPAR) and matrix metalloproteinase 2 and 9 (MMP2 and 9) signaling pathway [13].

The heat shock 70 kDa protein 5 (*HSPA5*), known as glucose regulated protein 78 (*GRP78*), also referred to as *BiP*, is traditionally regarded as a resident molecule in the endoplasmic reticulum (ER) and functions as a molecular chaperone involved in the unfolded protein response [14]. Beyond its chaperoning function, several studies show that HSPA5 is a multifunctional protein and exerts critical roles in cell proliferation, apoptosis and resistance to chemotherapy agents [15, 16]. Moreover, recent reports indicate that HSPA5 enhances cells invasion, migration and metastasis in colorectal and breast cancer [17–19]. In addition, elevated HSPA5 expression is linked to increased lymph node metastasis and poor prognosis in gastric cancer [20]. Conversely, another study have reported that downregulation of HSPA5 is found to enhance cancer metastasis in hepatocellular carcinoma [21]. In normal cells, HSPA5 is predominantly located in the ER. However, in tumor cells, HSPA5 is not only expressed in the ER but also at an elevated level on the cell surface of many tumors [22]. Moreover, HSPA5 expression on the cell surface of colon cancer is correlated with a poor prognosis [23].

The hypoxic microenvironment in tissues is a typical hallmark of solid tumors. ER stress induced by hypoxia promotes cellular tolerance to hypoxic stress [24]. In addition, ER stress is dysregulated in many types of cancer and contributes to cancer cell migration and invasion [25]. However, the mechanisms remain largely unknown. Previous studies have shown that induction of FOXM1 expression is mediated by hypoxia-inducible factor 1α (HIF-1α) in responses to hypoxia [26]. Moreover, the FOXM1 transcription factor which is over-expressed in many human cancers, is implicated in cancer cells invasion and metastasis [4]. Therefore, we investigated the relationship between FOXM1 with ER stress in colorectal cancer. In the present study, HSPA5 and spliced XBP1 were used as markers for ER stress. *FOXM1* mRNA level was firstly found to positively correlate with *HSPA5* in colorectal cancer and adjacent normal tissue samples. However, no significant correlation between *FOXM1* and spliced *XBP1* mRNA levels was found. Theses results suggested FOXM1 correlated with HSPA5 in colorectal cancer was not associated with ER stress. Subsequently, we provided evidences that FOXM1 increased HSPA5 transcription by binding to and stimulating HSPA5 promoter. Several studies have shown that FOXM1 is an important inducing factor of colorectal cancer cell migration and invasion [13]. Additionally, upregulation of HSPA5 also accelerates colorectal cancer cell migration and invasion [18]. Therefore, we investigated whether HSPA5 contributed colorectal cancer cells invasion and migration induced by FOXM1. Here, we found that enhancement of migration and invasion by FOXM1 was significantly attenuated by depletion of HSPA5 in colorectal cancer cell. Furthermore, FOXM1 triggered colorectal cancer cell migration and invasion were involved in activities of cell-surface HSPA5. Lastly, our results suggested FOXM1 facilitated the activities of MMP2 and 9 associated with HSPA5 in colorectal cancer cells.

## RESULTS

### *FOXM1* mRNA expression is elevated in most colorectal cancer tissues and positively correlated with *HSPA5*

**Figure 1:**
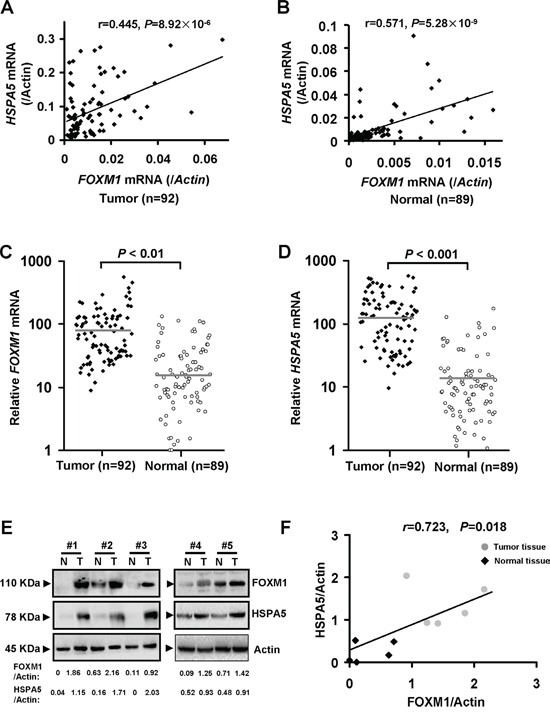
*FOXM1* mRNA expression is elevated in most colorectal cancer tissues and positively correlated with *HSPA5* **A.** and **B.** A moderately significant positive correlation was found between *FOXM1* and *HSPA5* mRNA expression values in colorectal tumor (n = 92, *r* = 0.445, *P* = 8.92×10^−6^) and corresponding adjacent normal tissues (n = 89, *r* = 0.571, *P* = 5.28×10^−9^). Expression of *FOXM1* and *HSPA5* were determined by qRT-PCR and normalized against *β-actin* (*Actin*) control. Pearson's correlation test was used to assess the difference and correlation between *FOXM1* and *HSPA5* mRNA expression. **C.** and **D.** The relative mRNA levels were expressed as fold increase relative to the lowest level after normalization to Actin. Unpaired two-sample *t* tests were used to compare the mean value for each gene between the tumor and normal samples. *P* values of <0.05 were considered significant. **E.** Protein expression of FOXM1 and HSPA5 was determined by way of Western blot analysis in colorectal tumor and corresponding adjacent normal tissue, Actin served as an internal control. All the gels were run under the same experimental conditions. Representative example of FOXM1 and HSPA5 expression in colorectal tumor tissues and adjacent normal tissues were shown. Bands were quantified using Image J software. **F.** A significant positive correlation was found between FOXM1 and HSPA5 protein expression values in colorectal tumor and corresponding adjacent normal tissue (n = 10, *r* = 0.723, *P* = 0.018).

To investigate the relationship between FOXM1 with ER stress in colorectal cancer, we first analyzed *HSPA5*, spliced *XBP1* and *FOXM1* mRNA expression by qRT-PCR in colorectal cancer specimens. A total of 92 colorectal cancer tissue specimens and 89 adjacent normal tissue specimens were included in this study. As shown in Figure 1A and 1B, we observed statistically significant positive correlations between *FOXM1* and *HSPA5* mRNA expression in colorectal cancer and adjacent normal tissue specimens (for tumor tissue: *r* = 0.445, *P* = 8.92×10^−6^; for normal tissue: *r* = 0.571, *P* = 5.28×10^−9^). Moreover, compared with adjacent normal tissue specimens, colorectal cancer tissue specimens exhibited higher *FOXM1* mRNA levels (Figure 1C). Similarly, Figure 1D indicated that the *HSPA5* mRNA levels in the colorectal cancer tissue samples were higher than the adjacent normal tissue specimens. In addition, Western blot analysis revealed that protein levels of FOXM1 and HSPA5 were upregulated in tumor samples relative to normal tissues (Figure 1E). Moreover, a statistically significant positive correlation between FOXM1 and HSPA5 protein levels was observed in these tissue specimens (Figure 1F, r = 0.723, *P* = 0.018). Notably, no significant correlations between *FOXM1* and spliced *XBP1* mRNA expression were found in colorectal cancer tissues (Supplementary Figure 1A, *r* = 0.036, *P* = 0.736). Additionally, we found statistically significant positive correlations between spliced *XBP1* and *HSPA5* mRNA expression in colorectal cancer (Supplementary Figure 1B, *r* = 0.443, *P* = 3.12×10^−6^).

### FOXM1 transcriptionally promotes HSPA5 expression in colorectal cancer cells

**Figure 2:**
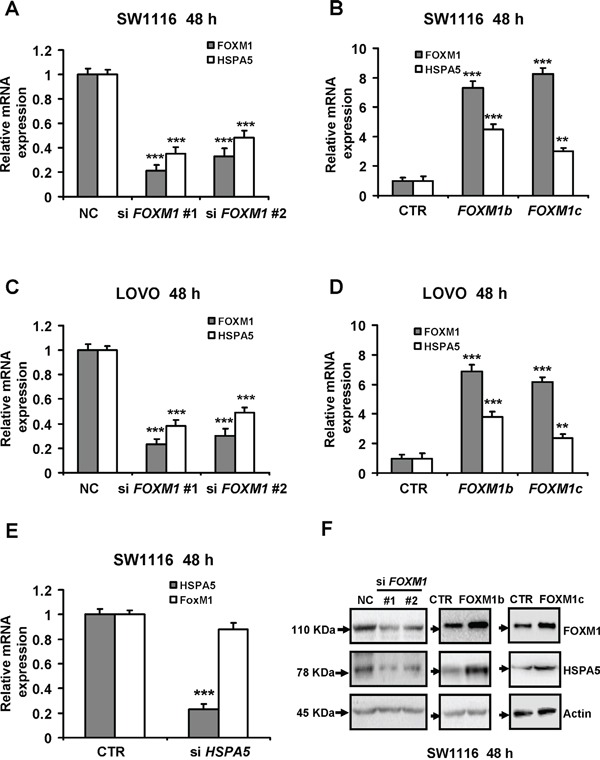
FOXM1 transcriptionally promotes HSPA5 expression in colorectal cancer cells **A.** and **C.** Two human colorectal cancer cell lines SW1116 and LOVO were treated with two *FOXM1* siRNAs (100 nM of si *FOXM1* #1 and si *FOXM1* #2 respectively) or 100 nM negative control (NC) siRNA for 48 h respectively. Expression of FOXM1 and HSPA5 were determined by qRT-PCR and normalized against β-actin (Actin) control. **B.** and **D.** SW1116 and LOVO cells were transfected with 3 μg FOXM1b and FOXM1c vectors for 48 h. Expression of FOXM1 and HSPA5 were determined by qRT-PCR and normalized against Actin control. **E.** SW1116 cells were treated with *HSPA5* siRNAs (100 nM) for 48 h. Expression of FOXM1 and HSPA5 were determined by qRT-PCR and normalized against Actin control. Results were the mean ± SD of triplicates, **, *P* < 0.01; ***, *P* < 0.001, compared with control. **F.** SW1116 cells were treated with the two *FOXM1* siRNAs (100 nM) or NC siRNA for 48 h respectively (left panel). SW1116 cells were transfected with 3 μg FOXM1b or FOXM1c vectors for 48 h (right panel). Cell lysates were subjected to Western blot analysis with the indicated antibodies.

To elucidate the relationship between FOXM1 and HSPA5 expression in colorectal cancer cells, we used two siRNAs against *FOXM1* as well as *FOXM1b* or *FOXM1c* lentiviral high-expression vectors to transfect two colorectal cancer cell lines, namely SW1116 and LOVO cells. For the effects of FOXM1 downregulation and overexpression were displayed clearly in Figure 2A and 2C. In the two cell lines, we found that reduction of FOXM1 by using siRNA also decreased HSPA5 expression; conversely, overexpression of FOXM1 markedly increased the expression of HSPA5 (Figure 2B and 2D). We next tested whether HSPA5 induced the expression of FOXM1 in colorectal cancer. We depleted HSPA5 using siRNA in SW1116 cells and found that knockdown of HSPA5 failed to decrease the mRNA expression of FOXM1 (Figure 2E). In addition, by Western blot analysis, we found knockdown of FOXM1 decreased the protein expression of HSPA5, whereas overexpression of FOXM1 using FOXM1b and FOXM1c expression vectors remarkably upregulated the protein levels of HSPA5 in SW1116 cells (Figure 2F).

### FOXM1 binds to and activates HSPA5 promoter

**Figure 3:**
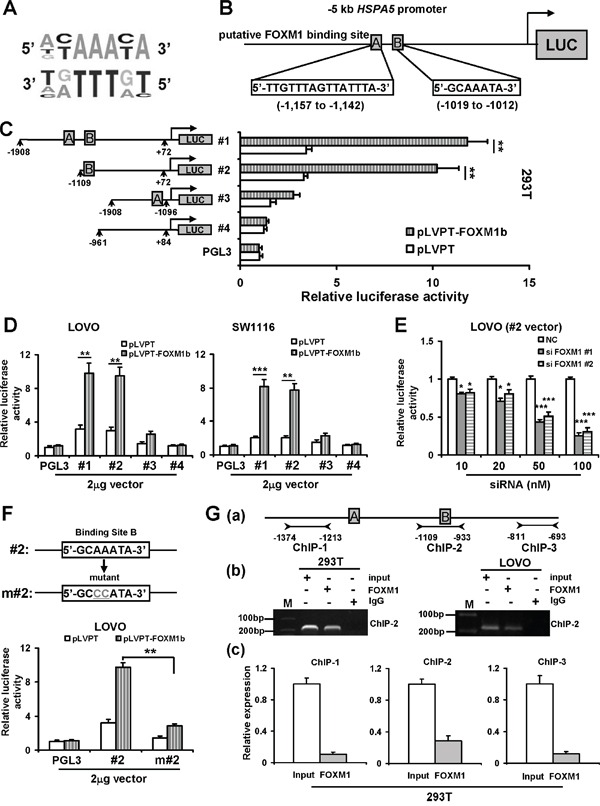
FOXM1 binds to and activates HSPA5 promoter **A.** The schematic representation of the FOXM1 DNA binding consensus sequence. **B.** The predicted positions of putative FOXM1 binding sites A and B in -2 kb HSPA5 promoter by gene sequence analysis. **C.** The schematic representation of the HSPA5 gene promoter deletion construction cloned into a firefly luciferase reporter vector pGL-3 (the numbers showed base pairs relative to the transcriptional start site of the *HSPA5* gene). The different region in -2 kb region of *HSPA5* promoter mediated the transcription activities of FOXM1 were determined using the Dual-Luciferase reporter assay system in 293T cells as described under materials and methods. **D.** The different reporter plasmid including different region of *HSPA5* promoter mediated the transcription activities of *FOXM1* were determined in LOVO and SW1116 cells. **E.** The #2 reporter plasmids were transfected into LOVO cells, followed by the transfection of increasing concentration *FOXM1* siRNA. After 48 h, the luciferase activities were measured. **F.** Mutational analysis of the FoxM1-binding site. The sequence of the putative FOXM1-binding site B was shown in both wild-type and mutant form. The #2 wide type and mutant reporter plasmid mediated the transcription activities of FOXM1 were determined in LOVO cells. **G. (a)** The positions of primers designed for ChIP assays. **(b)** and **(c)** ChIP assays were used to show direct binding of FOXM1 to endogenous *HSPA5* promoter regions. The chromatin of 293T and LOVO cells were cross-linked, sonicated and immunoprecipitated (IP) with either FOXM1 or control IgG antibody. The amount of promoter DNA associated with the IP chromatin was quantitated by PCR and qRT-PCR with primers specific to different *HSPA5* promoter regions. Results were the mean ± SD of triplicates, *, *P* < 0.05, **, *P* < 0.01; ***, *P* < 0.001, compared with control.

To determine whether HSPA5 was a potential downstream target of FOXM1, we scanned -2000 bp promoter region of human *HSPA5* gene with the canonical RYAAAYA forkhead binding motifs (Figure 3A) [27], and found two FOXM1 putative binding sites at the regions of -1157 to -1142 bp, -1019 to -1012 in the *HSPA5* promoter (Figure 3B). To confirm the direct association of FOXM1 with the *HSPA5* promoter, several truncated DNA fragments of the putative promoter region were amplified and cloned into a firefly luciferase reporter vector pGL-3 respectively. The luciferase reporter plasmids harboring different length of *HSPA5* promoter regions were named #1, #2, #3, #4 (Figure 3C). These reporter plasmids combined with FOXM1 expression vector were cotransfected into 293T cells. After treatment for 48 h, luciferase activity was detected. As shown in Figure 3C, #1 and #2 promoter plasmids driven luciferase activities were significantly higher than other promoter plasmids. Similar results were found in colorectal cancer LOVO and SW1116 cells (Figure 3D). Conversely, to measure the effect of decreased FOXM1 level on *HSPA5* transcription, we inhibited *FOXM1* gene expression by RNA interference in LOVO cells, and then transfected #2 promoter plasmid into the cells. As shown in Figure 3E, *FOXM1* siRNA obviously attenuated the luciferase activity driven by the #2 promoter plasmid in LOVO cells. These results showed that FOXM1 was associated with the transactivation of *HSPA5* through the promoter region from -1109 to -961 bp including site B.

To further evaluate the functional role of the putative FOXM1-binding site B in HSPA5 regulation, we conducted site-directed mutagenesis within the site B (Figure 3F). As shown in Figure 3F, m#2 construct exerted significantly much lower luciferase activity compared with that wild type #2 construct. To confirm the direct association of FOXM1 with the *HSPA5* promoter, we performed a ChIP assay in 293T and LOVO cells with a pairs of primers (named ChIP2) covering -1109 to -993 bp of *HSPA5* promoter. ChIP results shown in Figure 3G (b) revealed that obvious binding activities of FOXM1 on the *HSPA5* promoter region. Moreover, in 293T, SW1116 and LOVO cells, the amounts of promoter DNA associated with the ChIP were quantitated by qRT-PCR with three pairs of primers (named ChIP1, ChIP2 and ChIP3 respectively) covering the *HSPA5* promoter region -1374 to -1213 bp (ChIP1), -1109 to -993 bp (ChIP2) and -811 to -693 bp (ChIP3). As shown in Figure 3G (c) and Supplementary Figure 2, we found the amount of PCR production was higher with ChIP2 primers compared with ChIP1 and ChIP3 primers in all three cell lines. These results suggested that endogenous FOXM1 probably directly bound the putative binding site B (region between -1019 and -1012 bp of the *HSPA5* promoter) in colorectal cancer cells. All above results indicated that FOXM1 binds to and activates *HSPA5* promoter in colorectal cancer cells.

### Depletion of HSPA5 attenuates cell migration and invasion induced by FOXM1

**Figure 4:**
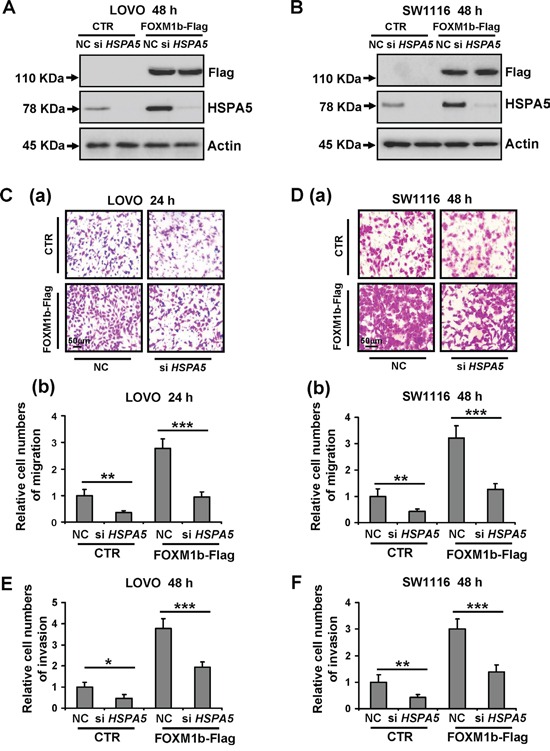
Depletion of HSPA5 attenuates cell migration and invasion induced by FOXM1 **A.** and **B.** LOVO and SW1116 cells were transfected with 3 μg of FOXM1b-Flag vectors for 8 h, and then treated with 100 nM NC (negative control) or *HSPA5* siRNA for additional 48 h. Cell lysates were subjected to Western blot analysis with the indicated antibodies. **C.** and **D.** LOVO and SW1116 cells were transfected with 3 μg of FOXM1b-Flag vectors for 8 h, and then treated with 100 nM NC or *HSPA5* siRNA for additional 8 h. **(a)** Cell migration of LOVO and SW1116 cells were assayed for indicated time by the transwell assay. **(b)** The statistical graph of relative cell migration changes was showed as fold increase relative to control after normalization to cell viability. **E.** and **F.** LOVO and SW1116 cells were transfected with 3 μg of FOXM1b-Flag vectors for 8 h, and then treated with 100 nM NC or *HSPA5* siRNA for additional 8 h. Cell invasion of LOVO and SW1116 cells were assayed for indicated time by matrigel-transwell assay. The statistical graph of relative cell invasion changes was showed as fold increase relative to control after normalization to cell viability. Results were the mean ± SD of triplicates, ***, *P* < 0.001, compared with control.

Several studies have shown that increased expression of FOXM1 induced colorectal cancer cell migration and invasion [13]. Additionally, HSPA5 also plays a major role in enhancing colorectal cancer cell migration [18]. Therefore, we next tested whether HSPA5 was required for FOXM1-driven cell migration. We ectopically introduced siRNA against *HSPA5* to FOXM1-overexpressed colorectal cancer cells respectively. As shown in Figure 4A and 4B, forced expression of FOXM1b in LOVO and SW1116 cells significantly increased FOXM1 protein expression, and *HSPA5* siRNA remarkably decreased HSPA5 protein expression. To exclude the possibility that the cell migration and invasion induced by FOXM1 or reduced by *HSPA5* siRNA were owing to cell proliferation, cell viability assay was performed simultaneously (Supplementary Figure 3). The statistical graph of relative cell migration and invasion changes was showed as fold increase relative to control after normalization to cell viability. Our results suggested the *FOXM1* and *HSPA5* siRNA decreased cell migration and invasion were independent of its cell proliferation regulatory function. As shown in Figure 4C–4F, after normalization to cell viability, FOXM1 significantly enhanced cell migration and invasion, however depletion of HSPA5 using siRNA obviously attenuated cell migration and invasion induced by FOXM1 in colorectal cancer cells. Similarly, we also found HSPA5 was required for FOXM1c-driven cell migration (Supplementary Figure 4). These above results provided evidence that FOXM1 induced colorectal cancer cells migration and invasion were partly dependent on HSPA5.

### Anti-HSPA5 antibody attenuates cell migration and invasion induced by FOXM1

**Figure 5:**
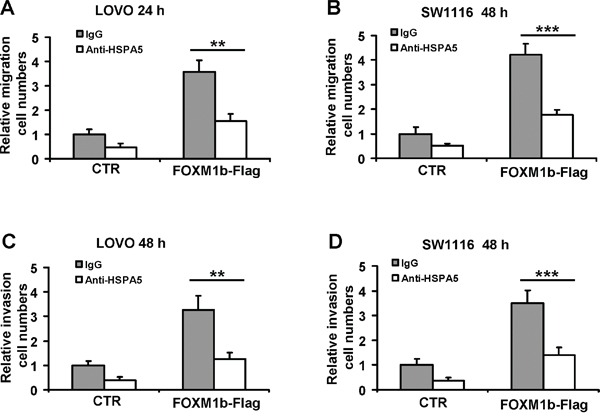
Anti-HSPA5 antibody attenuates cell migration and invasion induced by FOXM1 **A.** and **B.** LOVO and SW1116 cells were transfected with 3 μg of FOXM1b-Flag vectors for 8 h, migration of LOVO and SW1116 cells were assayed for indicated time by transwell assay in the presence of 10 μg/mL of normal IgG protein or anti-HSPA5 antibody. **C.** and **D.** LOVO and SW1116 cells were transfected with 3 μg of FOXM1b-Flag vectors for 8 h, invasion of LOVO and SW1116 cells were assayed for indicated time by matrigel-transwell assay in the presence of 10 μg/mL of normal IgG protein or anti-HSPA5 antibody. The statistical graphs of relative cell migration and invasion changes of results were showed. Results were the mean ± SD of triplicates, **, *P* < 0.01; ***, *P* < 0.001, compared with control.

Previous studies have shown that cell-surface HSPA5 increased colorectal cancer cell migration and invasion [18]. Hence, we detected whether cell-surface HSPA5 is required for colorectal cancer cell migration and invasion induced by FOXM1. LOVO and SW1116 cells transfected with FOXM1b overexpression construct were next treated with the HSPA5 antibody (10 μg/mL). Cell migration, invasion and viability assay were performed simultaneously (Figure 5 and Supplementary Figure 5). The statistical graph of relative cell migration and invasion changes was showed as fold increase relative to control after normalization to cell viability. Our results suggested FOXM1 induced and HSPA5 antibody reduced cell migration and invasion were independent of its cell proliferation regulatory function. As shown in Figure 5A and 5B, after normalization to cell viability, blocking cell-surface HSPA5 with antibody significantly diminished colorectal cancer cell migration. Similarly, matrigel-transwell assay showed that the cell numbers of invasion were obviously decreased (Figure 5C and 5D). These results indicated cell-surface HSPA5 was involved in colorectal cancer cell migration and invasion triggered by FOXM1.

### MMP2 and MMP9 expression and activities increased by FOXM1 are involved in cell-surface HSPA5

**Figure 6:**
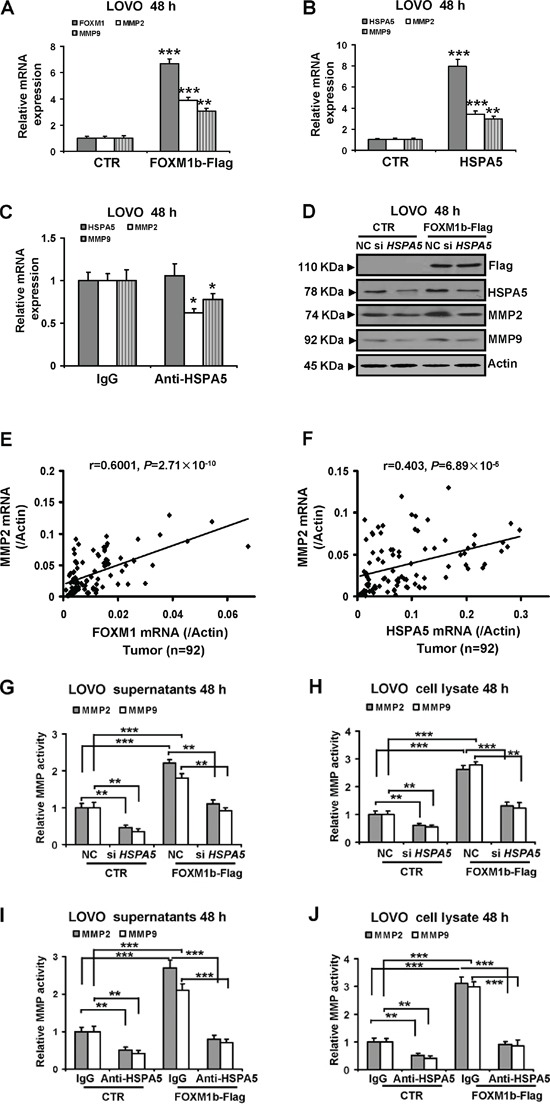
MMP2 and MMP9 expression and activities increased by FOXM1 are involved in cell-surface HSPA5 **A.** and **B.** LOVO cells were transfected with 3 μg FOXM1b and HSPA5 vectors for 48 h respectively. Expression of *FOXM1*, *MMP2*, *MMP9* and *HSPA5* were determined by qRT-PCR and normalized against actin control. **C.** LOVO cells were treated with 10 μg/mL of normal IgG protein or anti-HSPA5 antibody for 48 h respectively. Expression of *MMP2*, *MMP9* and *HSPA5* were determined by qRT-PCR and normalized against actin control. **D.** LOVO cells were transfected with 3 μg of FOXM1b-Flag vectors for 8 h, and then treated with 100 nM NC (negative control) or *HSPA5* siRNA for additional 48 h. Cell lysates were subjected to Western blot analysis with the indicated antibodies. **E.** and **F.** A significant positive correlation was found between FOXM1 and MMP2 expression values in colorectal tumor (n = 92, *r* = 0.6001, *P* = 2.71×10^−10^), between HSPA5 and MMP2 expression values in colorectal tumor (n =92, *r* = 0.403, *P* = 6.89×10^−5^). Expression of *FOXM1*, *HSPA5* and *MMP2* were determined by qRT-PCR and normalized against *β-actin* (*Actin*) control. Pearson's correlation test was used to assess the difference and correlation between *FOXM1* and *HSPA5* mRNA expression. **G.** and **H.** The MMP2 and MMP9 activities of media supernatants and cell lysates in LOVO cells were detected as described under materias and methods. **I.** and **J.** LOVO cells were transfected with 3 μg of FOXM1b-Flag vectors for 8 h, and then with 10 μg/mL of normal IgG protein or anti-HSPA5 antibody for additional 48 h respectively. The MMP2 and MMP9 activities of media supernatants and cell lysates in LOVO cells were detected as described under materias and methods. The statistical graphs of relative MMP2 and MMP9 activities changes of results were showed. Results were the mean ± SD of triplicates, **, *P* < 0.01; ***, *P* < 0.001, compared with control.

Aberrant FOXM1 expression enhances the migration and invasion of cancer cells by upregulating the transcription levels of *MMP2* and *MMP9* [28]. To determine whether FOXM1 increased MMP2 and MMP9 expression were involved in HSPA5, both *MMP2* and *MMP9* mRNA levels were analysed in FOXM1- and HSPA5-overexpressed LOVO cells by qRT-PCR. As shown in Figure 6A, the *MMP2* and *MMP9* mRNA expression levels in FOXM1-overexpressed LOVO cells were markedly increased compared with the control LOVO cells. Similarly, HSPA5 also significantly increased the mRNA levels of *MMP2* and *MMP9* in LOVO cells (Figure 6B). We further examined the effects of cell-surface HSPA5 on *MMP2* and *MMP9* mRNA levels. Results indicated that inhibition of cell-surface HSPA5 using antibody decreased the mRNA levels of *MMP2* and *MMP9* (Figure 6C). Moreover, LOVO cells transfected with FOXM1b overexpression construct were next treated with the *HSPA5* siRNA, we found knockdown of HSPA5 significantly attenuated protein expression of MMP2 and MMP9 induced by FOXM1 (Figure 6D). Moreover, we observed statistically significant positive correlations between *FOXM1* and *MMP2* mRNA expression (Figure 6E, n = 92, *r* = 0.6001, *P* = 2.71×10^−10^), between *HSPA5* and *MMP2* (Figure 6F, n = 92, *r* = 0.403, *P* = 6.89×10^−5^) in colorectal cancer tissue specimens. Recent study has documented that overexpression of HSPA5 enhances the JNK activities [29]. Additionally, the transcription of *MMP2* and *MMP9* is increased by activated through JNK-c-Jun signal pathway [30, 31]. To investigate whether HSPA5 increased MMP2 mRNA production was involved in JNK activities in colorectal cancer cells, HSPA5-overexpressed LOVO cells were treated with JNK inhibitor SP600125. We found SP600125 obviously suppressed *MMP2* mRNA levels induced by HSPA5 (Supplementary Figure 6).

Generation of activated forms of MMP2 and MMP9 was associated with cancer cell migration and invasion *in vitro* and *in vivo* [30]. Previous studies showed that FOXM1 promotes cancer cell migration and invasion by increasing the activities of MMP2 and MMP9 [28]. To identify whether HSPA5 was involved in FOXM1-activated MMP2 and MMP9 activities, we examined the activities in LOVO cells lysates and conditioned media (derived from LOVO cells after 48 h incubation). As show in Figure 6G–6J, the activities of MMP2 and MMP9 were remarkably increased by FOXM1. However, inductions of MMP activities by FOXM1 were partly blocked by *HSPA5* siRNA and HSPA5 antibody (Figure 6G–6J). The results pointed to FOXM1 facilitated the activities of MMP2 and MMP9 at least partly dependent on cell-surface HSPA in colorectal cancer cells.

## DISCUSSION

A series of studies *in vitro* and *in vivo* revealed that FOXM1 possesses a tumour-stimulative function and displays high expression in several cancer types including colorectal cancer [32, 33]. In this study, consistent with previous reports, we also found that *FOXM1* mRNA and protein levels were higher in colorectal cancer than in adjacent normal tissue. HSPA5, a molecular chaperone, could be induced under ER stress [34, 35]. Notably, HSPA5 has been reported to be overexpressed in several cancer types, contributing to the acquisition of several phenotypic malignant tumor hallmarks [22, 36]. Here, HSPA5 also was shown to be frequently highly expressed in colorectal cancer tissue, whereas lower levels HSPA5 were found in normal tissue. These results suggested HSPA5 was associated with tumorigensis in colorectal cancer.

In addition, transcriptional levels of *HSPA5* are promoted by ER stress induced by hypoxia, a condition frequently occured in the cores of solid tumors [24]. Previous studies have also shown that induction of FOXM1 expression is mediated by HIF-1α in responses to hypoxia. In this study, a significant positive correlation of *FOXM1* with *HSPA5* mRNA expression was observed in colorectal cancer specimens. To exclude the possibility of transcriptional upregulation of both *HSPA5* and *FOXM1* induced by hypoxia in advanced colorectal cancer, we analyzed the correlation between *FOXM1* and spliced *XBP1* mRNA, an indicator of ER stress. Notably, no correlation between *FOXM1* and spliced *XBP1* mRNA was found in the colorectal cancer tissues. However, *HSPA5* positively correlated with spliced *XBP1* mRNA in the colorectal cancer tissues. More importantly, a significantly positive correlation between *HSPA5* and *FOXM1* mRNA levels was found in normal colorectral tissues in which cells are generally under normoxia. All above results pointed out FOXM1 correlated with HSPA5 in colorectal cancer was independent of hypoxia or ER stress by hypoxia. Subsequently, in colorectal cancer cell lines, we showed that knocking down FOXM1 decreased HSPA5 expression, whereas overexpression of FOXM1 increased HSPA5 level. Mechanistically, we found FOXM1 enhanced the *HSPA5* gene at the transcriptional level through directly binding on the specific site in the promoter of *HSPA5*.

As described previously, FOXM1 has three isoforms FOXM1a, b and c due to differential splicing of exons [37]. FOXM1b and c function as transcriptional activators. Studies show FOXM1b and c are the predominant isoforms overexpressed in cancer cells, and play critical roles in the regulation of cell proliferation, apoptosis, migration and invasion [38]. In the present study, we also showed that both FOXM1b and c exaggerated colorectal cancer cells migration and invasion. Moreover, we found the mRNA expression of *HSPA5* in colorectal cancer cells was induced by both FOXM1b and c.

Cell metastasis and invasion have been regarded to be two important characteristics of cancer. FOXM1 is generally high-expressed in human cancers and exerts a critical role in cancer invasion and metastasis [4]. Increased expression of HSPA5 protein has been found in several types of cancer including colorectal cancer [39]. However, the functions of HSPA5 expression in the cells invasion and migration of various cancer types are controversial. Previous studies show that HSPA5 inhibits cell migration and invasion in hepatocellular carcinoma [40], whereas HSPA5 promotes colorectal and breast cancer cells invasion and migration *in vitro* [18, 19]. Notably, HSPA5 expression in cancer specimens is negatively correlated with lymphatic invasion in colorectal cancer patients [41]. Cell surface HSPA5 has been shown to bind activated alpha2-macroglobulin and activate PAK-2/LIMK/cofilin pathway, leading to increase prostate cancer cells migration [17, 42]. In addition, association of cell surface GRP78 with alpha2-macroglobulin facilitates the invasion and metastasis of hepatocellular carcinoma cells by promoting the interaction between EGFR and c-Src [43]. A recent study further showed that STAT3 mediates cell surface HSPA5-induced human breast cancer MCF-7 cells migration [19]. In this study, our results were consistent with previous reports that HSPA5 induced colorectal cancer cell migration and invasion in *vitro*. Moreover, we demonstrated that HSPA5 is required for FOXM1-induced migration and invasion of CRC cells since these effects were markedly attenuated by anti-HSPA5 antibody in LOVO and SW1116 cells. Together, these results suggested that the cell surface HSPA5 played an important role in FOXM1-triggered migration and invasion of CRC cells.

Additionally, it has been reported that FOXM1 increases the metastatic potential of colorectal cancer by positively regulating multiple invasion and metastasis associated molecules, which are involved in the degradation of extra cellular matrix components and angiogenesis such as uPA, uPAR, MMP2, MMP9 and vascular endothelial growth factor (VEGF) [3]. Moreover, previous study indicates that FOXM1 directly binds to and activates the promoter of the *MMP2* gene in glioma cells [28]. In the present study, we also indicated that the mRNA and protein levels of both *MMP2* and *MMP9* were enhanced by FOXM1 in LOVO cells. In addition, our data indicated that the expressions of MMP2 and MMP9 were obviously increased in LOVO cells transfected using *HSPA5* vectors; conversely they were markedly decreased by *HSPA5* siRNA. Moreover, we observed statistically significantly positive correlations between *FOXM1* and *MMP2* mRNA expression, between HSPA5 and MMP2 in colorectal cancer tissue specimens. Furthermore, our results suggested that FOXM1 increased the expression of MMP2 and MMP9 were involved in HSPA5. Recent study has documented that overexpression of HSPA5 enhances the JNK activities [29]. Additionally, the transcription of *MMP2* and *MMP9* is increased by activated through JNK-c-Jun signal pathway [30, 31]. Consistent with previous studies, we showed that the HSPA5 increased *MMP2* mRNA production involved in JNK activities in colorectal cancer cells. Tumor cells are reported to secret huge amounts of MMP2 and MMP9 in a paracrine or autocrine manner to stimulate their invasion and migration. Therefore, the activities of MMP2 and MMP9 were examined in the conditioned media and cell lysate of LOVO cells. Our data indicated that the activities of MMP2 and MMP9 in both media supernatants and cell lysate were obviously increased in LOVO cells transfected using FOXM1b vectors. However, the activities of MMP2 and MMP9 were markedly attenuated by *HSPA5* siRNA or HSPA5 antibody. These results suggested that HSPA5 were required for upregulation of the MMP2 and MMP9 activities induced by FOXM1.

In summary, this study provided mechanistic evidences to support the positive regulatory function of FOXM1 in HSPA5 expression. These findings were further supported by the evidence that FOXM1 expression in colorectal tumors and normal tissue from patients was positively correlated with HSPA5 expression. Moreover, we showed the critical role of this novel FOXM1-HSPA5 signaling in colon cancer migration and invasion. Based on the observations from our study, we suggested that aberrant FOXM1-HSPA5 signaling might be considered as a novel molecular target for designing novel therapeutic regimen to control colorectal cancer metastasis and progression.

## MATERIALS AND METHODS

### Sample information

Some samples were collected from 68 patients with colorectal cancer from the Affiliated Luoyang Central Hospital of Zhengzhou University (Zhengzhou, China), between 2007 and 2010, in which 65 patients contributed paired samples (i.e., both colorectal cancer tissue and adjacent normal tumor tissues located 5 cm from the edge of the tumor), and the other 3 patients contributed colorectal tumor tissue only. Other 24 pairs of samples were collected from the Boji-Affiliated Hospital (Zengcheng People's Hospital) of Sun Yat-Sen University (Guangzhou, China), between 2009 and 2010. All of the samples were collected within 15 minutes after the surgery and immediately frozen at 80°C. This study was approved by the Clinical Ethics Review Board at the Luoyang Central Hospital (IRB approved number: 2007101201) and the Boji-Affiliated Hospital of Sun Yat-Sen University (IRB approved number: A2009-05-08) and written informed consents were from all patients at their recruitment time.

### Cell culture and reagents

The colon cancer-derived cell lines SW1116 and LOVO were obtained from the American Type Culture Collection (ATCC, Manassas, VA, USA) and grown in DMEM medium (Gibco; Life Technologies, Carlsbad, CA) supplemented with 10% (v/v) fetal bovine serum (FBS) (Gibco; Life Technologies, Carlsbad, CA) at 37°C in 5% CO_2_ incubator. Cells were grown in monolayer and passaged routinely 2-3 times a week [44]. Dimethyl sulfoxide (DMSO), SP600125 and Crystal Violet was purchased from Sigma (St. Louis, MO, USA).

### Real-time reverse transcription PCR

Total RNA was extracted from colorectal cancer cell lines and patients tissue using TRIzol reagent (Invitrogen). Following DNaseI treatment, 2 μg of total RNA was reverse transcribed using cDNA synthesis kit (BioRad) to synthesize cDNA specimens. And then, quantitative real-time PCR (qRT-PCR) analysis of gene expression was performed using 2 μL of cDNA and SYBR Green Supermix (BioRad) as recommended by the manufacturer. For the primers, *FOXM1*: 5′-ACGTCCCCAAGCCAGGCTC-3′ (sense) and 5′-CTACTGTAGCTCAGGAATAA-3′ (antisense) [5]; *HSPA5*: 5′-CCAAGAGAGGGTTCTTGAATCTCG-3′ (sense), 5′-ATGGGCCAGCCTGGATATACAACA-3′ (antisense) [45]; Spliced *XBP1*: 5′-TGCTGAGTCCGCAGCAGGTG-3′ (sense), Spliced *XBP1*: 5′-GCTGGCAGGCTCTGGGGAAG-3′ (antisense) [46]; *MMP2*: 5′-CTCATCGCAGATGCCTGGAA-3′ (sense), *MMP2*: CAGCCTAGCCAGTCGGATTTG (antisense); *MMP9*: 5′-ACGCACGACGTCTTCCAGTA-3′ (sense), *MMP9*: 5′-CCACCTGGTTCAACTCACTCC-3′ (antisense);

*β-actin*: 5′-TTCTACAATGAGCTGCGTGTG-3′ (sense) and 5′-GGGGTGTTGAAGGTCTCAAA-3′ (antisense). *β-actin* was used as an internal control to normalize gene expression values for each gene expression analysis. The PCR was run in triplicate at 95°C for 2 min followed by 40 cycles of 95°C for 15 s, 56°C for 20 s, and 72°C for 20 s. Comparative quantification was performed using the 2^−ΔΔCt^ method. Each sample was analyzed in triplicate.

### Western blot analysis

Total proteins were isolated from cells with lysis buffer (50 mM Tris, pH 7.5; 150 mM NaCl; 1% NP40; 2.5 mM sodium pyrophosphate; 0.02% sodium azide; 1 mM EGTA, 1 mM EDTA; 1 mM b-glycerophosphate; 1 mM Na_3_VO_4_; 1 mM PMSF; 1 μg/mL leupeptin). The lysates were centrifuged at 12,000 rpm for 30 min at 4°C. The protein concentration was determined by Bradford dye method. Equal amounts (20 to 50 μg) of cell extract were subjected to electrophoresis in 6-15% sodium dodecyl sulfate-polyacrylamide (SDS-PAGE) and transferred to PVDF or nitrocellulose membranes (Millipore, Darmstadt, Germany) for antibody blotting. The membranes were blocked and then incubated with Flag, HSPA5, JNK1/2, p-JNK1/2 and β-actin antibodies (all from Cell Signaling Technologies, Massachusetts, USA), MMP2 and MMP9 (all from Abcam), FOXM1 (C-20) (Santa Cruz Biotech) overnight at 4°C. Subsequently, the membranes were incubated with a HRP-conjugated anti-mouse or -rabbit secondary antibody (Protein Tech Group, Chicago, IL) at room temperature for 1 h. The protein bands were visualized using an enhanced chemiluminescence reagent (ECL) kit (GE Healthcare; Munich, Germany), according to the manufacturer's instructions [47]. Bands were quantified using Image J software (Fujifilm, Tokyo, Japan) and normalized to a loading control.

### Cell invasion assay

Cell invasion assay was performed in 24-well transwell chambers (Costar, Cambridge, MA, USA) containing polycarbonate filters with 8-μm pores coated with Matrigel™ (1 mg/mL, BD Sciences, San Jose, CA, USA). Briefly, 3 × 10^4^ cells in 500 μL of serum-free medium were seeded into the upper chamber, and the lower chamber was filled with 800 μL of medium containing 10% FBS. After a 24-48 h incubation, the non-invasive cells were removed with a cotton swab, the number of cells that had invaded through the basement membrane were counted following staining with crystal violet solution. Finally, the cell numbers were counted and averaged in six random fields at a magnification of 100 ×.

### Cell migration assay

Cell migration assay was performed using Boyden Chambers (Transwell Costar, 6.5-mm diameter, 8-μm pore size) according to the manufacturer's instructions. Briefly, 3 × 10^4^ cells were resuspended in 500 μL serum-free medium and seeded into the upper chambe. Meanwhile, 800 μL of medium containing 10% FBS was added into the lower chamber. Cells were allowed to migrate for 24-48 h. Migrated cells were dyed with 1% crystal violet before observed. The cell numbers were counted under microscope. For each chamber, six fields of view were randomly selected for cell counting [48].

### Plasmids and siRNAs transfection

The plasmids encoding human *FOXM1* with flag tag and *HSPA5* were generated by PCR amplification. *FOXM1* was subcloned into the EcoRI and XhoI sites of a lentivirus vector (pLVPT) [49]. *HSPA5* was subcloned into the XhoI and HindIII sites of pLVPT. Cells were transfected with pLVPT-FOXM1, pLVPT-HSPA5 and the corresponding empty vector. In brief, cells were seeded at a density of 8 × 10^5^ cells per well in 6 well plate. After 8 h, 2 μg plasmid and 5 μL lipofectamine 2000 (Life Technologies, Carlsbad, CA) were diluted in 0.5 mL Opti-MEM medium respectively, and incubated at room temperature for 5 min. Diluted plasmid was mixed with the lipofectamine 2000 solution and then incubated for additional 25 min. Subsequently, cells were placed in the mixture with 2 μg plasmid and 5 μL lipofectamine 2000. After 8 h of transfection, 1 mL of complete culture medium was replaced, and experiments were conducted [50].

Small interfering RNAs (siRNAs) for down-regulating gene expression were done by transfection of RNA oligonucleotides with lipofectamine 2000 (Invitrogen, USA) according to the manufacturer's instructions. One day before transfection, cells were plated on a 6-well (35 mm) tissue culture plate in DMEM complete medium. After cells reached 30% confluence, they were transfected with siRNAs as previously described [51]. Briefly, cells were placed in 1 mL of siRNA mixture with 50 or 100 nM siRNA and 2.5 or 5 μL lipofectamine 2000 respectively. After 8 h of transfection, the medium with siRNA and lipofectamine 2000 were replaced with 2 mL of DMEM complete medium, and experiments were conducted 48 h after transfection. Protein levels were analyzed by Western blot. The negative control (NC) siRNA and siRNAs against *FOXM1* or *HSPA5* were synthesized by Shanghai GenePharma Co. For *FOXM1*:#1: 5′-CUCUUCUCCCUCAGAUAUAdTdT-3′ [52]; #2: 5′-GGACCACUUUCCCUACU dTdT-3′; For *HSPA5*: 5′- AAGGTTACCCATGCAGTTGTT-3′ [53]; the scrambled siRNA sequence was 5′-AAGGTGGTTGTTTTGTTCACT-3′.

### MMP2 and MMP9 activity assay

MMP2 and MMP9 activities were measured by SensoLyte 520 MMP2 and MMP9 Assay (fluorimetric) kit (ANASPEC, Fremont, CA). Briefly, the supernatants were collected from the cell culture plate and cells were lysed according to the manufacturer's protocol. And then the supernatants or cell lysates were incubated with 4-aminophenylmercuric acetate (AMPA) at a final concentration of 1 mM in the assay buffer for 1 h (MMP2 activity assay) and 2 h (MMP9 activity assay) at 37°C. Next, the supernatants or cell lysates treated with AMPA were mixed with MMP2 or MMP9 substrate by shaking the plate gently for 30 sec. The fluorescence intensity representing the MMP2 and MMP9 activity were measured at Ex./Em. wave lengths of 490 nm/520 nm wavelength.

### Promoter reporters and dual-luciferase assay

For generation of promoter reporters, the human *HSPA5* promoter regions were PCR amplified from genomic DNA of LOVO cells with the following primers:#1: -1908bp *HSPA5* promoter MluI, 5′-CGACGCGTCAGAGCTTTGCCCTTTCCAT-3′ (forward) and +72 bp *HSPA5* promoter BglII: 5′-GGAAGATCTCGAAACACCCCAATAGGTCA-3′ (backward); #2: -1109 bp *HSPA5* promoter MluI, 5′-CGACGCGTACGGGAAATGCCTACCGAG-3′ (forward) and +72 bp *HSPA5* promoter BglII: 5′-GGAAGATCTCGAAACACCCCAATAGGTCA-3′ (backward); #3: -1908 bp *HSPA5* promoter MluI, 5′-CGACGCGTCAGAGCTTTGCCCTTTCCAT-3′ (forward) and -1096 bp *HSPA5* promoter BglII: 5′-GGAAGATCTGTCTTGACAGTTCAATACTCGGT-3′ (backward); #4: -961 bp *HSPA5* promoter MluI: 5′-CGACGCGTCAAGACGATTTTCGCCTATCA-3′ (forward) and +84 bp *HSPA5* promoter BglII: 5′-GGAAGATCTCTCTCACACTCGCGAAACAC-3′ (backward). The mutant of the *HSPA5* promoter constructs were generated using the QuikChange7 site-directed mutagenesis kit (Stratagene, La Jolla, CA, USA) following the manufacturer's protocol. The PCR products were cloned into the corresponding restriction endonuclease Mlu1 and BglII sites of the pGL-3 basic Luciferase vector (Promega).

For the dual-luciferase assays, 293T or colorectal cancer cells were cotransfected with 2 μg of either *FOXM1*-Flag expression or control pLVPT vectors and 2 μg of the luciferase reporter constructs containing different HSPA5 promoter regions using Lipo2000 (Invitrogen). Cells were cultured for 48 h after cotransfection. Next cell lysates were used to analyse luciferase reporter gene expression using the dual-luciferase reporter assay system (Promega). Luciferase activities were normalized to the cotransfected pRL-TK plasmid. All experiments were conducted at least twice, in triplicate.

### Chromatin immunoprecipitation assays

Chromatin immunoprecipitation (ChIP) assays were performed according to a standard procedure with the following modifications. For immunoprecipitation, 2 mL of rabbit anti-FOXM1 antibody and control IgG was used. Immunoprecipitated DNA was ethanol precipitated and resuspended in 20 mL of double-distilled water. Total input samples were resuspended in 100 mL double-distilled water and diluted 1:100 before PCR reaction. The purified ChIP DNA sample and total input DNA sample were subjected to PCR and qRT-PCR (SYBR Green Supermix) analysis with the following primers: ChIP-1: -1374 bp forward: 5′-TGGTGCGTGCCTGTAATCCCA-3′ and -1213 bp backward: 5′-AACCAGGTGGAGTTCACTCT-3′; and ChIP-2: -1109 bp forward: 5′-ACGGGAAATGCCTACCGAG-3′ and -933 bp backward: 5′- TGATGTACACGCAGACAA-3′; ChIP-3: forward -811 bp 5′-GGAAAGTAAAGGGTGGCAAGC-3′ and -693 bp backward: 5′-ACGGTAAGTCGCAGGACAAG-3′. The PCR products were separated on 2% agarose gels and analyzed by ethidium bromide staining. All ChIP assays were conducted at least thrice and produced similar results.

### Statistics

All experiments were repeated three times and were expressed as mean ± standard deviation (SD). *P* values were calculated using student's *t* test and *P* value < 0.05 was considered significant. Pearson's correlation coefficient was calculated to analyse the correlation between *FOXM1* and *HSPA5* mRNA expression in colorectal cancer and normal tissues. Statistical analysis was analysed using the Statistical Package for Social Sciences (SPSS) software (version 16.0).

## SUPPLEMENTARY DATA FIGURES



## Abbreviations

HSPA5: heat shock 70 kDa protein 5
MMP: matrix metalloproteinase
EMT: epithelial-to-mesenchymal transition
uPAR: urokinase-type plasminogen activator receptor
GRP78: glucose regulated protein 78
ER: endoplasmic reticulum
HIF-1α: hypoxia-inducible factor 1α
FBS: fetal bovine serum
ChIP: Chromatin immunoprecipitation
siRNA: small interfering RNA
VEGF: vascular endothelial growth factor
