# Supplementary material for: FOXM1 promotes invasion and migration of colorectal cancer cells partially dependent on HSPA5 transactivation
Source: Oncotarget. 2016 Mar 28;7(18):26480–95. doi: 10.18632/oncotarget.8419 (PMC5041994; doi:10.18632/oncotarget.8419)
Supplement: Supplementary file 1 [file oncotarget-07-26480-s001.pdf]

SUPPLEMENTARY DATA

MATERIALS AND METHODS

Determination of cell viability

CCK8 assay was conducted to assess the cell viability according to the manufacturer's instructions (Roche Diagnostics, Mannheim, Germany). Briefly, cells were plated into 96-well plates at a density of 0.5-1×10^4 cells per well and incubated for at least 8 h in a 5% CO_2 atmosphere at 37 °C before exposure to drugs. The media were then removed, and cells were treated with drugs. After the cells were incubated for indicated time, CCK8 reagents were added to each well and the plate was incubated for another 2 h at 37 °C. Absorbance of the media was then measured using a Micro-plate Reader (Bio-Rad, Hercules, CA) at 450 nm. This assay was conducted in triplicate.

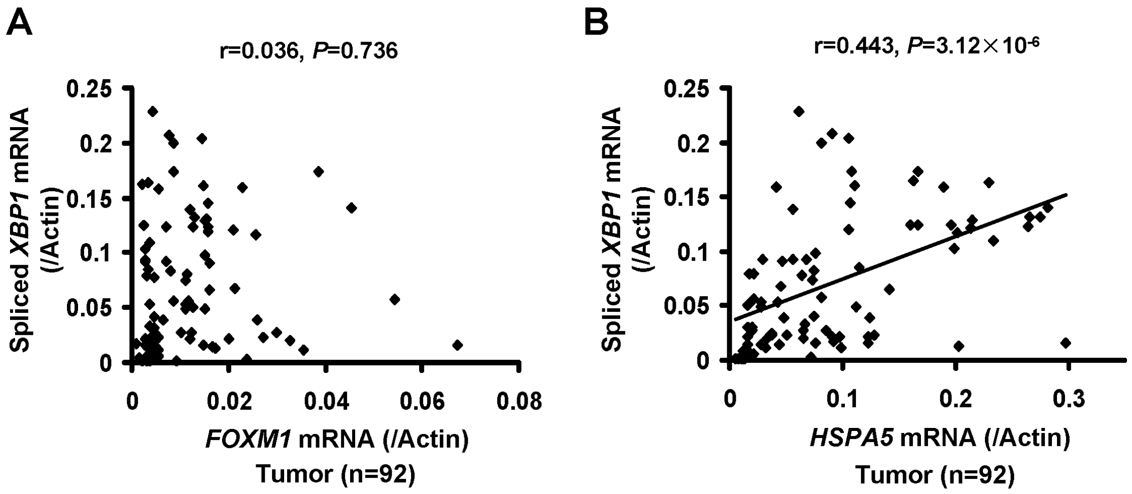

Figure 1: The correlation between *FOXM1*, *HSPA5* and spliced *XBP1* expression in colorectal cancer tissues. A. No significant correlation was found between *FOXM1* and spliced *XBP1* expression values in colorectal tumor tissues (n = 92, $r = 0.036$, $P = 0.736$). **B.** A moderately significant positive correlation was found between *HSPA5* and spliced *XBP1* expression values in colorectal tumor tissues (n = 92, $r = 0.443$, $P = 3.12\times10^{-6}$). Expression of *FOXM1*, spliced *XBP1* and *HSPA5* were determined by qRT-PCR and normalized against β-actin (Actin) control. Pearson's correlation test was used to assess the correlation between *FOXM1*, *HSPA5* and spliced *XBP1* mRNA expression.

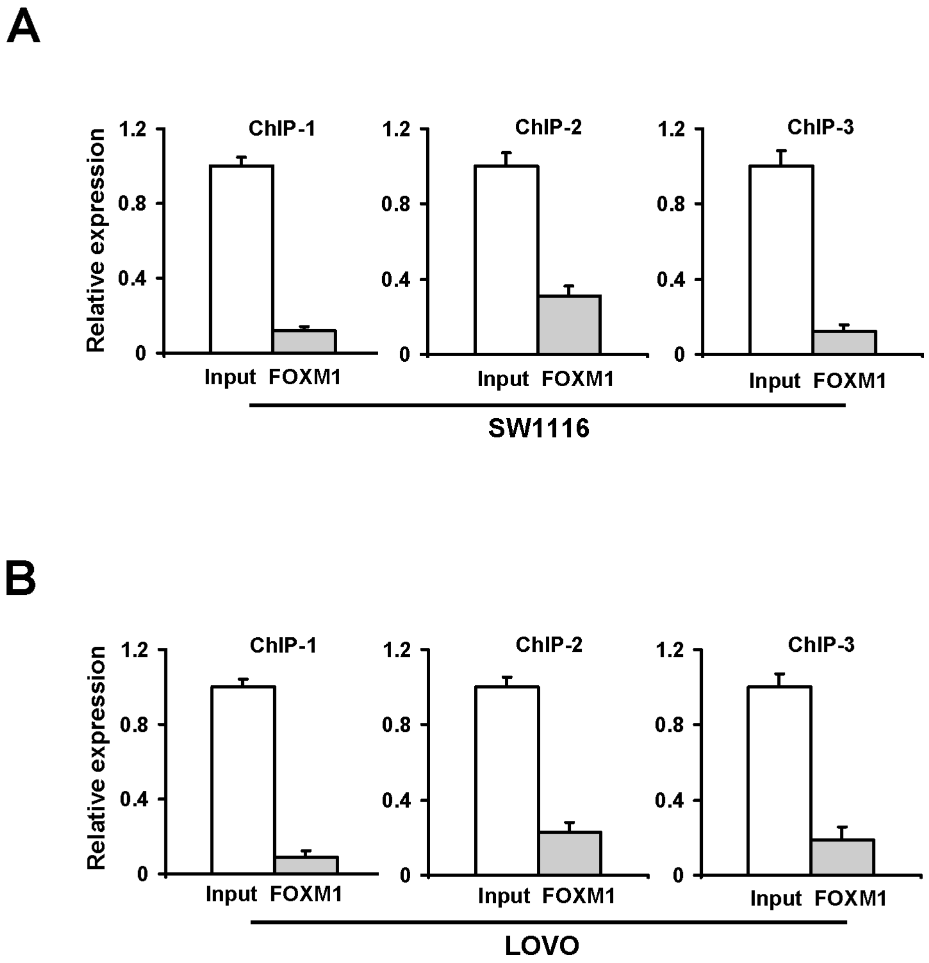

Figure 2: FOXM1 binds to HSPA5 promoter in SW1116 and LOVO cells. A. and **B.** ChIP assays were used to show direct binding of FOXM1 to endogenous *HSPA5* promoter regions. The chromatin of SW1116 and LOVO cells were cross-linked, sonicated and immunoprecipitated with either FOXM1 or control IgG antibody. The amount of promoter DNA associated with the IP chromatin was quantitated by qRT-PCR with primers specific to different *HSPA5* promoter regions as described in Figure 3. Results were the mean ± SD of triplicates.

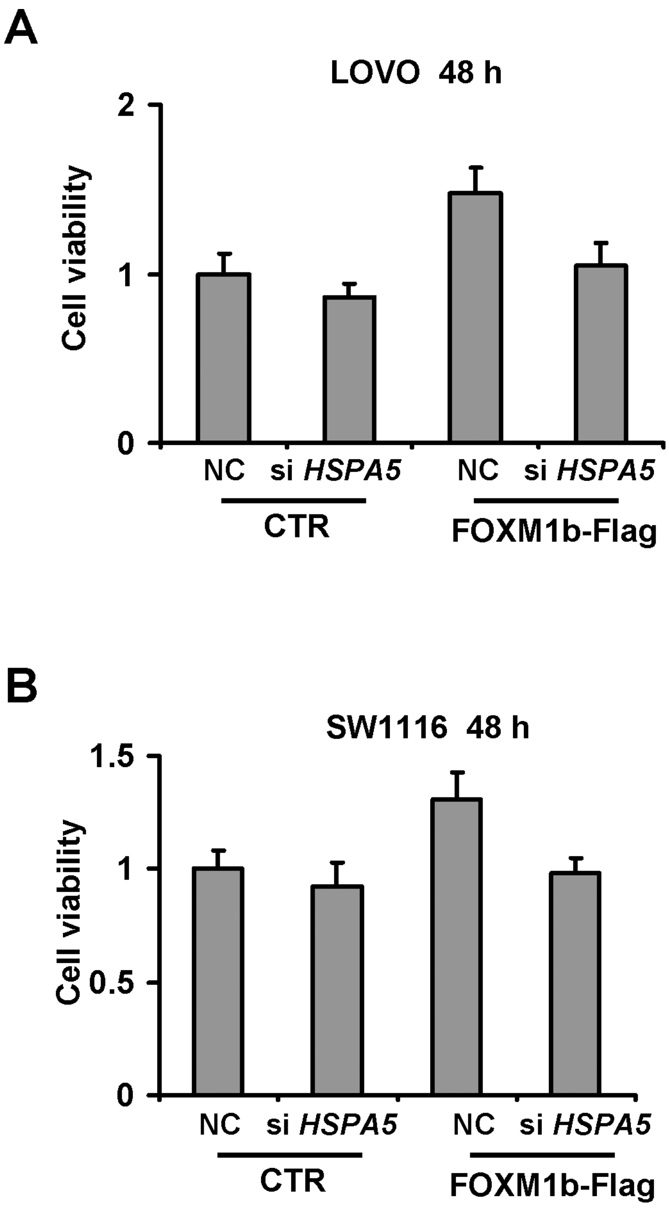

Figure 3: Depletion of HSPA5 attenuates cell viability induced by FOXM1b in colorectal cancer cells. A. and **B.** LOVO and SW1116 cells were transfected with 3 mg of FOXM1b-Flag vectors for 8 h, and then treated with 100 nM NC (negative control) and HSPA5 siRNA for additional 48 h. Cell viability was detected by CCK8 assay. Results were the mean ± SD of triplicates.

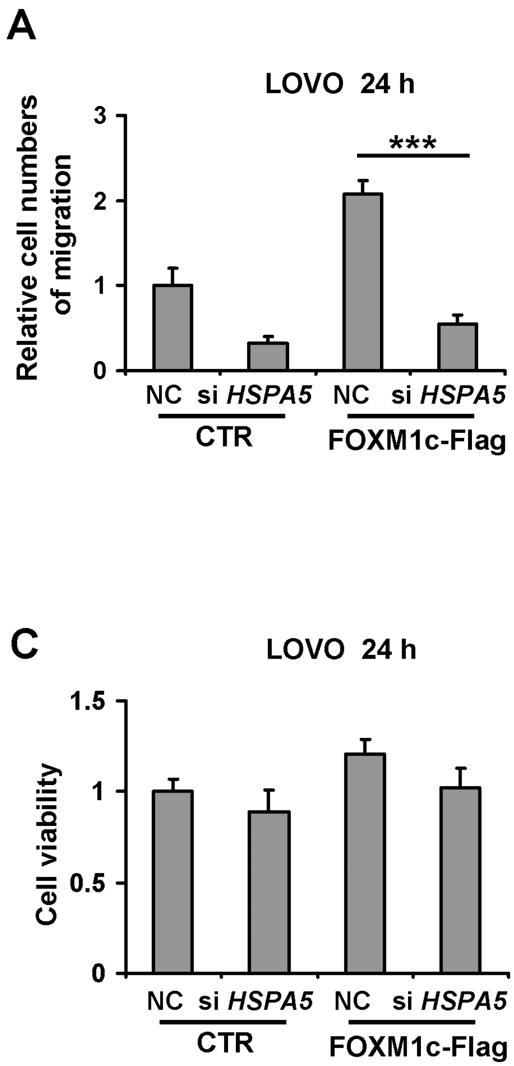

Figure 4: HSPA5 is required for FOXM1c-driven cell migration in colorectal cancer cells. LOVO and SW1116 cells were transfected with 3 mg of FOXM1b-Flag vectors for 8 h, and then treated with 100 nM NC (negative control) and *HSPA5* siRNA for additional 8 h. **A.** and **B.** Cell migration of LOVO and SW1116 cells were assayed for indicated time by transwell assay. The statistical graph of relative cell migration changes was showed as fold increase relative to control after normalization to cell viability. **C.** and **D.** Cell viability was detected for indicated time by CCK8 assay. The statistical graphs of relative cell viability changes were showed. Results were the mean ± SD of triplicates, ***, $P < 0.001$, compared with control.

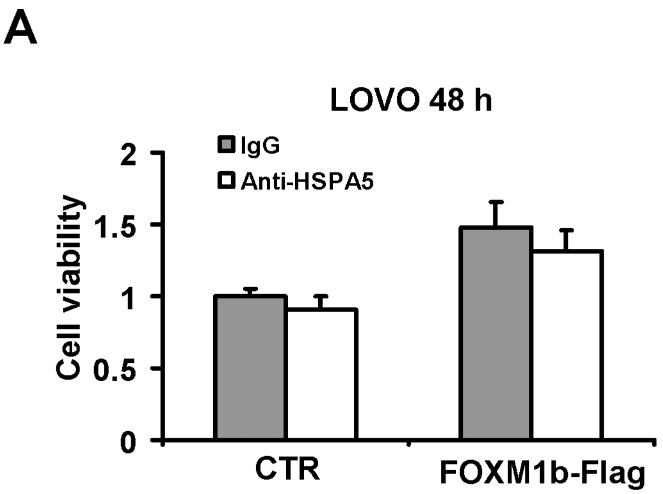

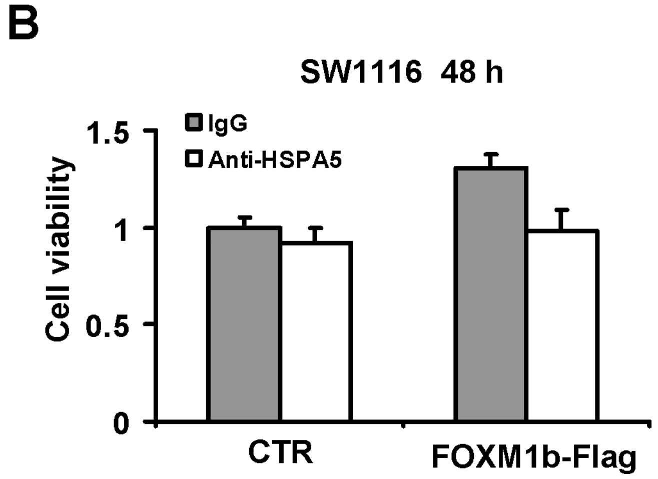

Figure 5: HSPA5 antibody attenuates cell viability induced by FOXM1 in colorectal cancer cells. A. and **B.** LOVO and SW1116 cells were transfected with 3 mg of FOXM1b-Flag vectors for 8 h, cell viability of LOVO and SW1116 cells were detected for indicated time by CCK8 assay in the presence of 10 mg/mL of normal IgG protein and anti-HSPA5 antibody. Results were the mean ± SD of triplicates.

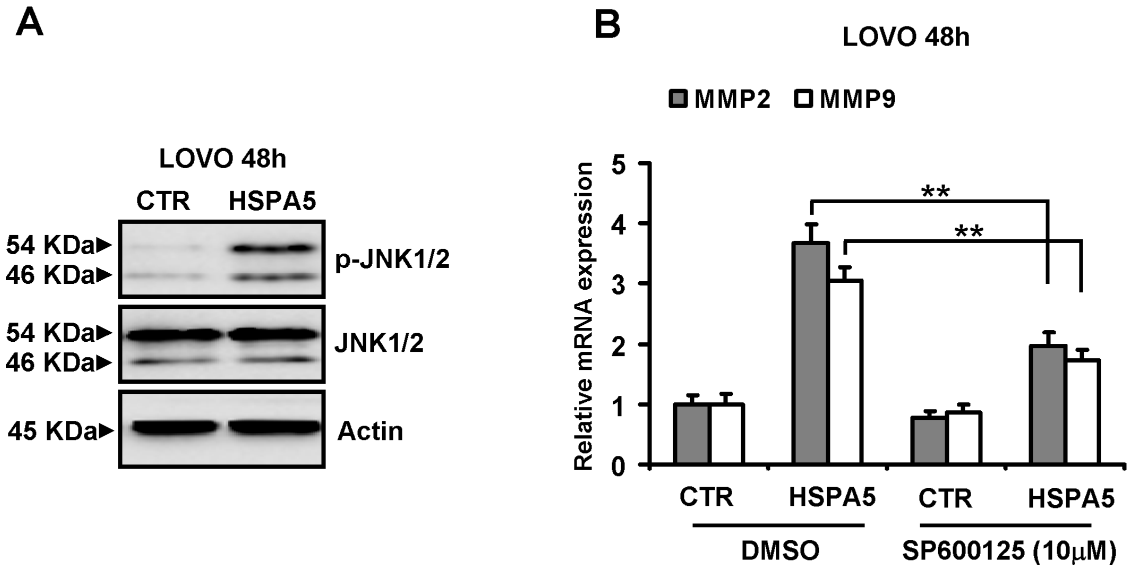

Figure 6: HSPA5 increased MMP2 levels is involved in JNK activities in LOVO cells. A. LOVO cells were transfected with 3 mg of HSPA5 vectors for 48 h. And then protein expression of JNK, phosphorylated JNK and HSPA5 was determined by Western blot analysis. **B.** LOVO cells were transfected with 3 mg of HSPA5 vectors for 6 h, and then were treated with SP600125 (10 mM) for additional 48 h. Expression of *MMP2* mRNA was determined by qRT-PCR and normalized against *β-actin* (*Actin*) control. Results were the mean ± SD of triplicates, **, $P < 0.01$, compared with control.
